# Prediction of DNA Integrity from Morphological Parameters Using a Single‐Sperm DNA Fragmentation Index Assay

**DOI:** 10.1002/advs.201900712

**Published:** 2019-05-24

**Authors:** Yihe Wang, Jason Riordon, Tian Kong, Yi Xu, Brian Nguyen, Junjie Zhong, Jae Bem You, Alexander Lagunov, Thomas G. Hannam, Keith Jarvi, David Sinton

**Affiliations:** ^1^ Department of Mechanical and Industrial Engineering University of Toronto 5 King's College Road Toronto Ontario M5S 3G8 Canada; ^2^ Hannam Fertility Centre 160 Bloor St. East Toronto Ontario M4W 3R2 Canada; ^3^ Department of Surgery Division of Urology Mount Sinai Hospital University of Toronto 60 Murray Street, 6th Floor Toronto Ontario M5T 3L9 Canada

**Keywords:** DNA integrity, hyaluronic acid, machine learning, single cell, sperm morphology

## Abstract

Intracytoplasmic sperm injection is a popular form of in vitro fertilization, where single sperm are selected by a clinician and injected into an egg. Whereas clinicians employ general morphology‐based guidelines to select the healthiest‐looking sperm, it remains unclear to what extent an individual sperm's physical parameters correlate with the quality of internal DNA cargo—a measurement that cannot be obtained without first damaging the sperm. Herein, a single‐cell DNA fragmentation index (DFI) assay is demonstrated, which combines the single‐cell nature of the acridine orange test with the quantitative aspect of the sperm chromatin structure assay, to create a database of DFI‐scored brightfield images. Two regression predictive models, linear and nonlinear regression, are used to quantify the correlations between individual sperm morphological parameters and DFI score (with model test *r* at 0.558 and 0.620 for linear and nonlinear regression models, respectively). The sample is also split into two categories of either relatively good or bad DFIs and a classification predictive model based on logistic regression is used to categorize sperm, resulting in a test accuracy of 0.827. Here, the first systematic study is presented on the correlation and prediction of sperm DNA integrity from morphological parameters at the single‐cell level.

## 1 Introduction

Male‐factor infertility is a global human health issue, affecting 25–50 million couples worldwide.^1^, ^2^, ^3^ Studies have shown that DNA integrity is an important predictor of fertility potential as evidenced by a significant correlation with fertility and embryonic development.^4^, ^5^, ^6^ In this regard, DNA integrity may be a better predictor of male fertility potential than conventional measurements of sperm morphology and motility.^7^, ^8^ Intracytoplasmic sperm injection (ICSI) is a form of in vitro fertilization that requires insertion of a single sperm into the cytoplasm of the oocyte, making it possible to fertilize an egg with a sperm that may not have otherwise reached or penetrated the oocyte naturally. In ICSI, a clinician assesses the suitability of each sperm using readily observable criteria, namely, sperm shape and motility.^9^, ^10^ However, clinicians are blind to a selected sperm's internal properties, such as DNA integrity, given that such assays are destructive and render the sperm nonviable.^11^ There is thus a need for a noninvasive means of predicting sperm DNA integrity to enhance the sperm selection process and guarantee that sperm with low DNA fragmentation are selected for ICSI. To what extent sperm physical properties may correlate (and be predictive of) sperm DNA integrity is the subject of ongoing research. Assessing morphology parameters at the single‐cell level is noninvasive but has not been correlated to DNA integrity.

Whereas many studies have suggested a correlation between morphology and DNA integrity at sample level,^12^, ^13^, ^14^, ^15^ others indicate that there is no such correlation.^16^ For those studies that do claim a correlation, there is no agreement on what specific sperm features (head size, head shape, vacuoles, etc.) are most relevant to predicting DNA integrity. For example, Tang et al.^12^ reported that sperm head shape and the presence of a bent neck correlate significantly with sperm DNA integrity, whereas Utsuno et al.^13^ reported that sperm head shape and vacuoles have significant correlations with sperm DNA integrity. In the work of Wilding et al.^14^ and Dariš et al.,^15^ other head abnormalities are significantly correlated with sperm DNA integrity. Whereas there is no collective agreement on what sperm morphological aspects are most relevant to DNA integrity at the sample level, even less is known at the single‐cell level.^17^ Any correlation between sperm morphology and DNA integrity at the single‐cell level has yet to be ascertained. Whereas sample‐level correlations are important in diagnosing infertility, single‐cell‐level correlations serve a different purpose in helping a clinician decide which sperm should be selected for ICSI. Therefore, the correlation at a single‐cell level will undoubtably assist in selecting single sperm for the success of ICSI. This work is the first systematic analysis of the correlation and prediction of sperm DNA integrity based on morphological parameters at the single‐cell level.

Sperm assessment assays have been developed for either morphology or DNA analysis. Sperm morphology can be observed from Papanicolaou,^18^ Shorr,^19^ and a rapid staining technique such as Diff‐Quik stain^20^ and Testsimplets.^21^ However, the air‐drying step inherent to these methods may change sperm morphology.^22^ Most importantly, the air‐drying method does not ensure strong cell attachment to the glass, as the cells only weakly adhere to the glass after drying, and often in positions that preclude morphological analysis. Sperm DNA integrity analysis assays, such as the quantitative sperm chromatin structure assay (SCSA), comet assay, and terminal deoxynucleotidyl transferase dUTP nick end labeling assay, have in turn offered a glimpse into a cell's internal DNA cargo. The SCSA is the most published and reproducible method.^23^ However, use of flow cytometry is incompatible with other methods, and thus a combined morphology and SCSA analysis is not feasible. A robust high throughput staining method that enables combined sperm morphology observation and DNA integrity analysis has not been reported.

In this work, we measure the correlation between sperm morphology and DNA integrity at the single‐cell level and establish a means of predicting DNA integrity from a cell's physical attributes. This work is the first systematic study on the correlation and prediction of sperm DNA integrity from morphological parameters at the single‐cell level. We demonstrate a single‐cell DNA fragmentation index (DFI) assay, which combines the best attributes of two existing sperm diagnostics assays—the microscope‐based acridine orange test and the quantitative SCSA. We demonstrate an effective sperm preparation method for concurrent brightfield and fluorescent imaging, which involves air‐drying on hyaluronic acid (HA) functionalized glass. The HA chemically immobilized on glass allows strong HA‐sperm adherence and enables combined analysis of sperm morphology and DNA integrity at the single‐cell level, and subsequent analysis of correlations and predictive capabilities. Furthermore, the HA‐functionalized glass can be used for selection of live sperm bound to the surface. Established sperm morphological parameters, such as sperm head circularity (*C*), head width (HW), head length, midpiece width (MW), acrosome area (AA), and acrosomal vacuole area (VA), are measured for each cell. The DNA integrity of each sperm cell, quantified in terms of DFI, is evaluated by measuring the fluorescence intensity of acridine orange (AO) bound to DNA. We show that the sperm circularity and acrosome area correlate to sperm DNA integrity (model Pearson's correlation coefficient *r* = 0.571) to a higher degree than other parameters in a linear regression. To predict single‐sperm DNA integrity from morphological parameters, we used two predictive models: regression and classification. Linear and nonlinear regression machine learning algorithms are used to predict single‐sperm DFI by using morphological parameters (model test *r* = 0.558 and 0.620 for linear and nonlinear regression, respectively). By applying logistic regression, we predict the categorical sperm DNA integrity from morphological parameters with a test accuracy at 0.854. Our work resolves the correlation between established morphological parameters and DNA integrity, and presents opportunities for improved clinical selection of sperm with the help of machine learning.

## 2 Results and Discussions

### 2.1 Preparation of Sperm for Morphology and DNA Integrity Analysis

**Figure 1:**
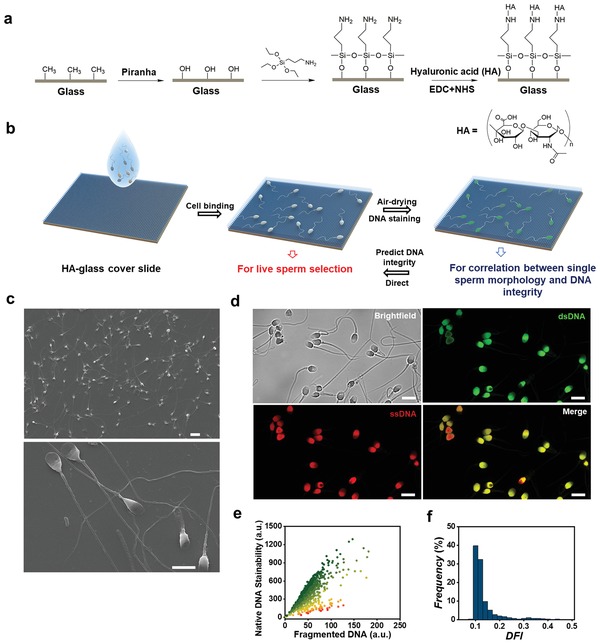
A sperm‐binding and air‐drying method to prepare sperm for morphological and DNA integrity analysis. a) Chemical functionalization of the glass cover slide with hyaluronic acid (HA). Glass is treated with (3‐aminopropyl)triethoxysilane (APTES) and then HA reacts with APTES by an EDC/NHS reaction. b) Schematic of sperm preparation for simultaneous imaging for morphological and DNA integrity analysis. A glass cover slide is functionalized with hyaluronic acid (HA). After HA binging and air drying, sperm were stained with acridine orange (AO) for quantification of dsDNA and ssDNA. c) Scanning electron microscope (SEM) images of sperm bound to HA‐glass cover slide. Scale bars are 10 and 5 µm for top and bottom images, respectively. d) Brightfield image (top left) and fluorescence images of identical sperm with stained dsDNA (top right) and ssDNA (bottom left), and merged image of sperm stained with dsDNA and ssDNA. Scale bars are 10 µm. e) Distribution of native DNA stainability against single‐sperm fragmented DNA. f) Distribution of single‐sperm DNA fragmentation index (DFI).

A glass cover slide was chemically functionalized with HA for sperm binding (**Figure** **1**a). The glass was first treated with piranha to generate hydroxyl group for the reaction with the (3‐aminopropyl)triethoxysilane (APTES). After silanization, HA was immobilized on the glass cover slide via an *N*‐(3‐dimethylaminopropyl)‐*N*′‐ethylcarbodiimide/*N*‐hydroxysuccinimide (EDC/NHS) reaction with the amine groups on glass. The chemical modification of glass with HA was confirmed by fluorescence imaging of Fluorescein isothiocyanate (FITC) labeled HA and by observing the change of hydrophobicity of glass (Figure S1, Supporting Information). After several washing steps, a drop of sperm suspension was placed on the HA‐functionalized glass cover slide (Figure 1b). After sperm settled down on the HA glass, mature and live sperm were bound to HA by the CD44 cell receptor,^24^, ^25^, ^26^, ^27^ while nonmotile sperm simply settled on the HA glass. An HA‐modified petri‐dish (PICSI dish) has been used in fertility clinics to select mature sperm.^28^, ^29^, ^30^ Unlike the physical absorption of HA hydrogel on a plastic substrate in PICSI dishes,^31^ we functionalized the glass surface with HA using permanent chemical bonding. While bound to HA, sperm heads lied flat on the HA glass with tails continuously beating (see Videos S1 and S2 in the Supporting Information). Then, the whole sample was dried in air until the water was completely evaporated (maximum of 5 min). Almost all sperm lie flat with their head attached to the HA glass (Figure 1c). Cell adhesion was sufficiently strong to withstand cell fixation with organic solvents, such as methanol and acetic acid. Figure 1c shows scanning electron microscopy (SEM) images of sperm bound to HA glass after fixation.

The sperm attached to the HA glass were stained with AO for the quantification of fragmented DNA for single‐sperm DFI. For AO staining, we followed the same protocol as SCSA,^32^ but used confocal microscopy rather than flow cytometry to image the cells. The AO stained the double‐stranded DNA (dsDNA) and single‐stranded DNA (ssDNA) such that they emit in the green and red, respectively, under excitation at 488 nm (Figure 1d). The value of DFI was calculated as the ratio of red fluorescence intensity to the sum of red and green fluorescence intensities. During drying and staining, sperm remain firmly adhered to HA glass. In addition, the process did not change sperm morphology, as confirmed by measurement of morphology parameters before and after drying and staining (Figure S2, Supporting Information), as the cells firmly bound to the HA on glass. In contrast, in other sperm morphological assays, air drying of the cells on bare glass may cause the cells to shrink. Each sperm was imaged using both brightfield and fluorescence for morphological assessment and DNA integrity analysis, respectively. A spinning disk confocal microscope was used with an overall magnification of 100 ×. To ensure consistent imaging conditions between experiments, we calibrated our imaging system using fluorescent microbeads, as shown in Figure S3 (Supporting Information).

Images of 1056 sperm from six donors were captured using brightfield and fluorescence imaging. Figure 1e shows the distribution of native DNA stainability (defined as the green fluorescence intensity of AO‐stained nuclei) against fragmented DNA stainability (defined as the red fluorescence intensity of AO‐stained nuclei) for individual sperm. A large portion of sperm (>80%) have a DFI in the range of 0.07–0.15 (Figure 1f). The distribution of DNA stainability against fragmented DNA and number distribution of DFI for individual samples are plotted in Figure S4 (Supporting Information).

### 2.2 Morphological Parameters of Single Sperm

**Figure 2:**
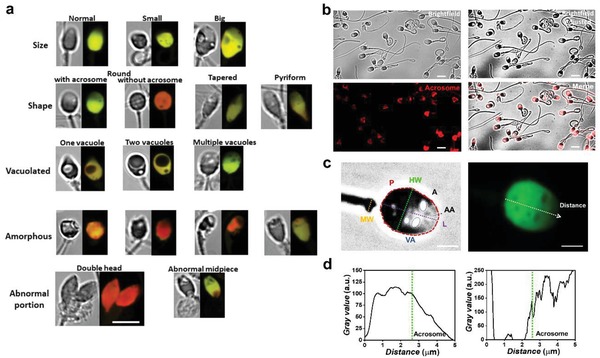
Single‐sperm morphologies. a) Morphological classification of sperm using brightfield and fluorescence imaging after acridine orange (AO) staining. Scale bar is 5 µm. b) The brightfield image of sperm (top left) was adjusted by enhancing contrast (top right). The fluorescence image (bottom left) of the sperm with stained acrosomes was merged with the adjusted brightfield image. Scale bars are 5 µm. c) Morphological parameters obtained from the brightfield image (left) of a sperm, including head area (*A*), head perimeter (*P*), head width (HW), head length (*L*), midpiece width (MW), acrosome area (AA), and vacuole area (VA). The same sperm stained with AO was imaged using fluorescence microscopy (right). Scale bars are 2 µm. d) Gray value along the major axis of the sperm for corresponding brightfield and fluorescence images in (c).

According to the WHO standard,^9^ sperm with different morphological features, including size, shape, vacuolation, and abnormal portions, can be identified from brightfield images (**Figure** **2**a). For clear visualization and quantification of acrosomes and vacuoles, we adjusted the contrast of the brightfield images (Figure 2b). The acrosomal region of individual sperm was confirmed by staining the acrosome with lectin peanut agglutinin (PNA) from *Arachis hypogaea* (peanut) conjugate (bright region of the sperm in the brightfield images in Figure 2b).

Several dimensional parameters, including head area (*A*), head perimeter (*P*), HW, MW, AA, and VA in acrosomal regions, were measured using the adjusted brightfield images (Figure 2c, left). The acrosome area was defined as the area of the sperm head where the gray value along the major axis sharply increases (Figure 2d, left). The proportion of the sperm head area with increased gray value in the brightfield image was consistent with the proportion of the head with decayed fluorescence intensity in the corresponding fluorescence image (Figure 2d). Likewise, the vacuole area in the sperm head in the brightfield image was consistent with the corresponding fluorescence image.

### 2.3 Correlation between Single‐Sperm Morphology and DNA Integrity and Prediction of DFI by a Linear Regression Model

After extraction of several morphological parameters from individual sperm, a secondary parameter was determined from these parameters: sperm head circularity. *C* is defined as

(1) $$C\;=\;\frac{4\pi A}{P^{2}}$$

To avoid collinearities between independent variables, we set *C*, HW, *L*, MW, VA, and AA as the independent variables, and Ln(DFI) as the dependent variable, and applied a linear regression model including main effects and two‐factor interactions. The weights of each independent variable and the two‐factor interaction terms, characterized as parameters, were estimated by a least square estimator. The estimated parameters and standard errors for each term are listed in **Table** **1**. The weight of *C* is the largest among all parameters, suggesting the maximum contribution of the sperm head circularity to the sperm DNA integrity in the linear regression. Pearson's correlation coefficient *r* was used to evaluate the goodness of linear correlation between the independent and dependent variables.^33^, ^34^ For 1056 sperm from the six donor samples, the *r* value is 0.571, suggesting a significant correlation between these morphological parameters with sperm DNA integrity.

**Table 1:** Statistical parameters from the linear regression model. The linear correlation equation is the sum of each term in the first column multiplied by the estimated parameter in the “Estimate” column. The standard error (Std error) of estimation, *t* ratio, *P* value, and variance inflation factor (VIF) are presented for each term

Term	Estimate	Std error	t ratio	Prob > |*t*|	VIF
Intercept	−1.59	0.17	−9.11	<.0001	–
*C*	−0.48	0.20	−2.41	0.02	1.82
VA	0.04	0.01	2.74	0.01	3.58
AA	−0.06	0.00	−19.18	<.0001	1.15
(*C*‐0.86097)*(*C*‐0.86097)	−5.16	1.33	−3.88	0.00	2.88
(*C*‐0.86097)*(*L*‐4.9618)	−0.39	0.16	−2.43	0.02	2.45
(MW‐1.32059)*(MW‐1.32059)	0.01	0.00	4.23	<.0001	1.03
(*C*‐0.86097)*(VA‐0.68287)	−0.40	0.16	−2.45	0.01	3.32
(*L*‐4.9618)*(VA‐0.68287)	−0.03	0.01	−2.46	0.01	3.78
(VA‐0.68287)*(VA‐0.68287)	−0.01	0.00	−2.09	0.04	4.36
(*C*‐0.86097)*(AA‐3.80839)	0.35	0.07	4.72	<.0001	2.39
(HW‐3.3639)*(AA‐3.80839)	−0.02	0.01	−2.42	0.02	1.48
(*L*‐4.9618)*(AA‐3.80839)	0.02	0.01	4.18	<.0001	2.11
(AA‐3.80839)*(AA‐3.80839)	0.01	0.00	7.39	<.0001	1.34

**Figure 3:**
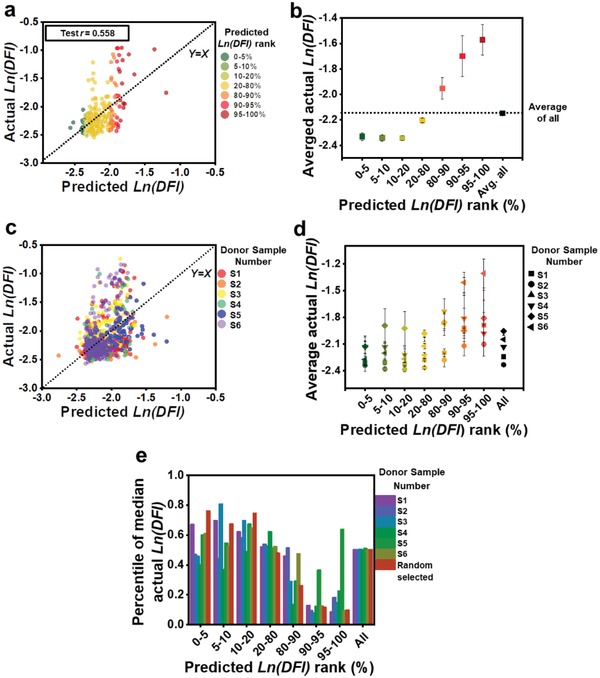
Correlation and prediction of single‐sperm DNA integrity by a linear regression model using morphological parameters. a) Actual Ln(DFI) plotted against predicted Ln(DFI) for the test set using a linear regression algorithm with a training set randomly selected across all donors. Pearson's correlation coefficient *r* is 0.558 for the test set. b) The average actual Ln(DFI) for each rank group of predicted Ln(DFI) for the test set in (a). c) Actual Ln(DFI) plotted against predicted Ln(DFI) for test sets consisting of individual donor samples using a linear regression algorithm with a training set randomly selected from all other donors. d) Actual Ln(DFI) plotted against predicted Ln(DFI) for each rank group for individual donor sample test sets in (c). e) The percentile of the median actual Ln(DFI) for each rank group of predicted Ln(DFI).

We developed a machine learning algorithm incorporating training, validation, and testing for prediction of single‐sperm DFI. The actual Ln(DFI) of individual sperms was randomly separated into three groups: training (65%), validation (10%), and testing (25%). **Figure** **3**a shows the distribution of actual Ln(DFI) plotted against the predicted Ln(DFI) for the testing set after training and validation by a linear regression model. The black dotted line represents the data where the actual Ln(DFI) equals the predicted Ln(DFI). For 1056 sperms from six donor samples, the *r* value is 0.565, 0.549, and 0.558 for the training, validation, and testing sets, respectively. The *P* value is an indicator of the significance (*P* < 0.05) of each term in the correlation analysis,^35^ as listed in Table 1. The measurement of multicollinearity for all independent variables, Variance Inflation Factor (VIF), was calculated for each case (Table 1). The values of VIF for all terms in the fitting equation were less than 10, indicating the noncollinearity of all terms.^35^

We ranked the predicted Ln(DFI) from low to high and distributed data into several sets by percentile: 0%–5%, 5%–10%, 10%–20%, 20%–80%, 80%–90%, 90%–95%, and 95%–100%. The residual (the difference between predicted Ln(DFI) and actual Ln(DFI)) for each data point increases as the predicted Ln(DFI) increases (Figure 3a). Figure 3b shows the averaged actual Ln(DFI) for each predicted Ln(DFI) set. The averaged actual Ln(DFI) increases as the predicted Ln(DFI) increases. For all sets, except the rank of 20%–80%, the difference between the actual Ln(DFI) in each rank and the averaged actual Ln(DFI) of all test data is significant, as indicated by *P* < 0.05 from a *t*‐test.

The linear regression model was also used to predict the Ln(DFI) of individual donor samples. Figure 3c shows the distribution of actual Ln(DFI) against its predicted Ln(DFI) for six donor samples. Each sample was set as the testing group and the rest of the samples were used for training and validation by a random selection ratio of 0.8:0.2. The distribution of each donor sample follows a similar trend: the residual between actual Ln(DFI) against predicted Ln(DFI) increases as the predicted Ln(DFI) increases. The test set *r* value for donor samples 1–6 was 0.458, 0.427, 0.509, 0.446, 0.099, and 0.513, respectively.

Figure 3d shows the average actual Ln(DFI) in each predicted Ln(DFI) rank for individual donor samples. The distribution of average actual Ln(DFI) for each sample follows a similar trend. The evaluation of significant difference between the actual Ln(DFI) in each rank and the averaged actual Ln(DFI) of all testing data is indicated by *P* value in Table S1 (Supporting Information).

From a clinical sperm selection perspective, sperm with high DNA integrity (low DFI) are of more interest than sperm with low DNA integrity (high DFI). Therefore, the comparison of the distribution of actual Ln(DFI) in each predicted Ln(DFI) ranks (in the lower range) to the actual Ln(DFI) for the full testing set is an indication of the enrichment of the predicted Ln(DFI) to the lower actual Ln(DFI). Figure 3e shows the overall percentile of the actual Ln(DFI) of median predicted Ln(DFI) in each predicted Ln(DFI) rank. For most of the predicted Ln(DFI) in the rank of 0%–20%, especially 10%–20%, the percentile of the actual Ln(DFI) of median predicted Ln(DFI) is significantly higher than the percentile of actual Ln(DFI) for the median predicted Ln(DFI) of all samples (50%).

The linear correlation between single‐sperm morphology and DNA integrity provides a comprehensive way to understand the importance of each morphological parameter to DNA integrity. The morphological parameters extracted from the high resolution brightfield images are all sperm‐inherent properties, and do not vary under different lighting conditions, e.g., light intensity and wavelength. Among all morphological parameters, the head circularity had the largest correlation with DFI. This finding is consistent with an earlier study which showed the effect of head shape on fertilization rate.^15^ However, there is no clear conclusion that sperm head shape is significantly correlated with DNA integrity yet.^36^ In addition, our work revealed the significance and weight of other morphological parameters, such as acrosome size and acrosomal vacuole size, in predicting DNA integrity.

### 2.4 Correlation between Single‐Sperm Morphology and DNA Integrity and Prediction of Sperm DFI by a Nonlinear Regression Model

**Figure 4:**
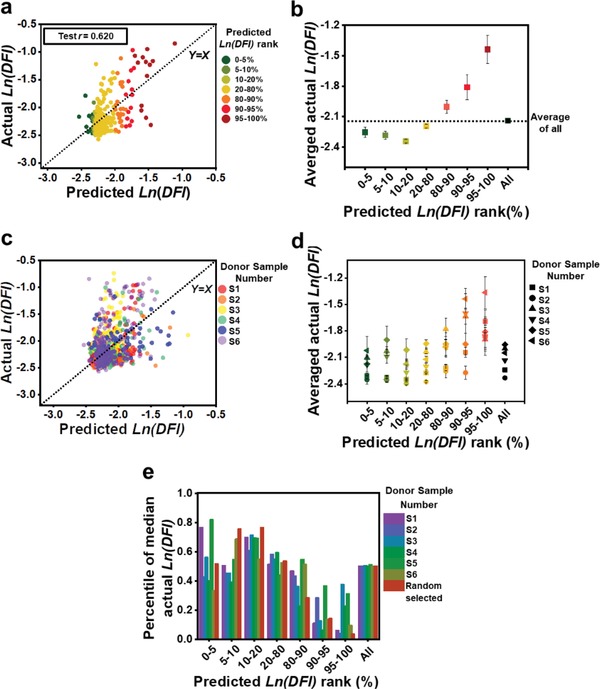
Correlation and prediction of single‐sperm DNA integrity by a nonlinear regression model using morphological parameters. a) Actual Ln(DFI) plotted against predicted Ln(DFI) for the test set using a nonlinear regression algorithm with a training set randomly selected across all donors. Pearson's correlation coefficient *r* is 0.620 for the test set. b) The average actual Ln(DFI) for each rank group of predicted Ln(DFI) for the test set in (a). c) Actual Ln(DFI) plotted against predicted Ln(DFI) for test sets consisting of individual donor samples using a nonlinear regression algorithm with a training set randomly selected from all other donors. d) Actual Ln(DFI) plotted against predicted Ln(DFI) for each rank group for individual donor sample test sets in (c). e) The percentile of the median actual Ln(DFI) for each rank group of predicted Ln(DFI).

Single‐sperm DFI was also predicted by a nonlinear regression machine learning model. In the nonlinear regression model, the morphological parameters of *C*, HW, *L*, MW, VA, and AA were set as the independent variables, and Ln(DFI) as the dependent variable, with a sigmoidal activation function and one hidden layer with 20 nodes. The full data set was split into training (65%), validation (10%), and testing (25%) groups. **Figure** **4**a shows the fit of actual Ln(DFI) to the predicted Ln(DFI) for the test set. The score *r* for training, validation, and test set is 0.630, 0.561, and 0.620, respectively.

The predicted Ln(DFI) for the testing set was also ordered from low to high. The residual between predicted Ln(DFI) and actual Ln(DFI) increased as the predicted Ln(DFI) increased (Figure 4a). Figure 4b shows the average actual Ln(DFI) for each percentile of predicted Ln(DFI). The actual Ln(DFI) for all sets, except the sets of 80%–90% group, is significantly different than the average actual predicted Ln(DFI) for all testing data. Figure 4c shows the actual Ln(DFI) plotted against its predicted Ln(DFI), for each donor sample, with *r* = 0.502, 0.502, 0.326, 0.477, 0.118, and 0.514 for donor samples 1–6, respectively. Figure 4d shows the average actual Ln(DFI) in each predicted Ln(DFI) rank for individual donor samples. The distribution of average actual Ln(DFI) for each sample follows a similar trend. The evaluation of significant difference between the actual Ln(DFI) in each rank and the averaged actual Ln(DFI) of all test data is indicated by *P* value in Table S1 (Supporting Information).

Figure 4e shows the overall percentile of the actual Ln(DFI) of median predicted Ln(DFI) in each predicted Ln(DFI) rank. For most of the predicted Ln(DFI) in the rank of 0%–20%, especially 10%–20%, there was significant enrichment of actual Ln(DFI) percentile as compared to the median of actual Ln(DFI) of all samples (50%).

### 2.5 Prediction of Categorical Sperm DNA Integrity Using Logistic Regression

**Figure 5:**
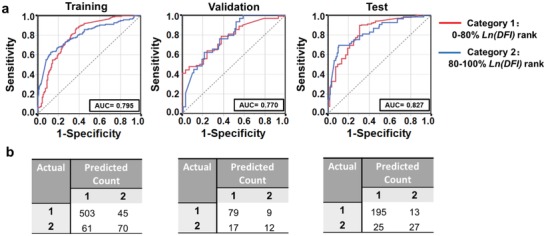
Performance of a logistic regression model in the prediction of single‐sperm DNA integrity using morphological parameters. a) The receiver operating characteristic (ROC) curves for the training, validation, and test set from data randomly selected across all donors using a logistic regression algorithm. Area under the curve (AUC) is 0.795, 0.770, and 0.827 for the training, validation, and test set, respectively. The outcome is classified into two categories: top 80% and bottom 20% Ln(DFI) rank. b) The confusion matrix for the training, validation, and test set in (a) for 1056 sperm.

After organizing sperm into one of two categories according to relative DFI (good vs bad), we used logistic regression to study to what extent these categories could be predicted based on morphology. Rather than evaluating a model's ability to “rank” sperm as before, we now evaluate a model's ability to “classify” sperm. Specifically, we ranked the Ln(DFI) from low to high, and split Ln(DFI) values into two categories, e.g., 0%–80% and 80%–100%. A logistic regression algorithm was used to train, validate, and test. **Figure** **5**a shows the receiver operating characteristic (ROC) curves for training, validation, and test data randomly selected across the six donor samples. The ROC curve represents how the true positive rate (Sensitivity) changes with the false positive rate (1‐Specificity) for different cut‐offs of the morphological parameters. The area under the curve (AUC) value indicates the ability of the algorithm to distinguish the two categories (e.g., 0%–80% and 80%–100% Ln(DFI)). For training, validation, and test sets, the AUC values are all greater than 0.75.

Figure 5b shows the confusion matrix of the training, validation, and test data sets obtained by logistic regression. The accuracy is 0.844, 0.778, and 0.854 for the training, validation, and test sets, respectively. For different classifications, most of the accuracy and AUC values are above 0.750 with few cases being in the range of 0.600–0.750. The accuracy and AUC values for different classifications (i.e., different good vs bad distributions) are listed in Table S2 (Supporting Information). Our use of a logistic regression model in classification shows that it is possible to predict single‐sperm categorical DFI at reasonable accuracy.

## 3 Conclusion

In ICSI, clinicians rely on empirical criteria such as sperm head shape for single‐sperm selection. Our findings provide a different perspective on single‐sperm selection for practical applications. First, the correlation between morphological parameters and sperm DNA integrity established in this work reveals the importance of each morphological parameter in the correlation with sperm DNA integrity. Our work is the first systematic study on the numerical correlation between morphology and DNA integrity at the single‐sperm level. The sperm head circularity has the strongest correlation with DNA integrity in our linear regression model. Second, the machine learning approach that made the prediction of single‐sperm DNA integrity possible also presents an opportunity to aid in clinical sperm selection. Specifically, by applying a logistic regression machine learning algorithm, we can classify sperm into good/bad categories with an accuracy as high as 85%. Third, in a clinical sperm selection context, the prediction of low DFI (high quality) sperm is especially important. A group of low DFI sperm, selected by the linear and nonlinear models as the predicted top 10%–20%, would be significantly enriched as compared to the bulk sample, with a median in the 50–70th percentile. Our prediction methods offer a means of predicting low DFI sperm and thus may give very important guidance to clinicians selecting sperm in fertility clinics.

We developed a high throughput and robust sperm binding approach for concurrent imaging of sperm morphology and DNA quality. The platform developed in this work to bind and image sperm could also be used for multipurpose staining, such as cell marker detection, tail beating profile, and chromosomal aneuploidy characterization—all of which would improve the predictive capability of the machine learning algorithm. In addition, the open‐air nature of the platform allows the retrieval of live sperm for subsequent analysis or injection. Live mature sperms can be trapped, imaged, and assessed after binding to the HA‐functionalized glass cover slide. There is precedent for the use of bound sperm cells in the clinical practice of ICSI.

Overall, the presented method of sperm binding and imaging allow high throughput and robust analysis of single‐sperm morphology and DNA integrity. The correlation established between sperm morphology and DNA integrity and prediction of DNA integrity by machine learning algorithms can give important guidance to clinicians on single‐sperm selection.

## 4 Experimental Section

*HA Functionalization of Glass*: A glass cover slide (50 mm × 24 mm, 0.16–0.19 mm thickness, VWR, USA) was treated with piranha solution (sulfuric acid: H_2_O_2_ = 3:1 by volume) for 30 min. Next, the glass cover slide was immersed in a solution of 10% v/v APTES (Sigma‐Aldrich, USA) in acetone at 60 °C for 30 min, rinsed with acetone, and dried in air. The glass slide was then heated to a temperature of 110 °C in an oven for 60 min. HA (Calbiochem, USA), *N*‐(3‐dimethylaminopropyl)‐*N*′‐ethylcarbodiimide hydrochloride (EDC·HCl, Sigma‐Aldrich, USA), and NHS (Sigma‐Aldrich, USA) were dissolved in 2‐(*N*‐morpholino)ethanesulfonic acid (MES) buffer (50 × 10^−3^ m, pH = 5, Bioshop Canada Inc., Canada) to a final concentration of 5 mg mL^−1^ and the solution was stirred for 1 h until the HA was completely dissolved. The silanized glass cover slide was treated with the above solution for 30 min and rinsed with water before the cell suspension was loaded onto the glass. The FITC‐labeled HA was purchased from Sigma‐Aldrich, USA.

*Sperm Sample Preparation and Acridine Orange Stain*: The donor semen samples were purchased from ReproMed Ltd., Canada with all donors signed and informed consent. The raw frozen semen sample was thawed in a 37 °C water bath for 30 min and purified by dispersing 200 µL raw semen into 2 mL of pure sperm wash medium (Nidacon, PSW‐100, Sweden) and centrifuging at 300 g for 5 min, with one additional wash. The final volume of sperm suspension was 50 µL. A reservoir made from binding a polydimethylsiloxane (PDMS, Dow Corning, USA) slab cut with a hole (8 mm in diameter) to the HA‐functionalized glass cover slide was used to contain cells and solutions. Before binding, the surfaces of the HA‐functionalized glass cover slide and PDMS slab were treated with plasma by a surface energy treater for 30 s. A 3 µL drop of purified sperm suspension was loaded into the reservoir on the HA‐functionalized glass cover slide. After binding to the glass, sperm were dried in air for 10 min until the liquid on the glass was completely evaporated. Then, 20 µL of TNE buffer (1 × 10^−3^ m Tris‐HCl, 15 × 10^−3^ m NaCl, and 0.1 × 10^−3^ m ethylenediaminetetraacetic acid (EDTA), pH = 7.4) was added to the reservoir. Sperm were treated with 40 µL of acid‐detergent solution (80 × 10^−3^ m HCl, 150 × 10^−3^ m NaCl, 1 mg mL^−1^ Triton X‐100, pH = 1.2) for 30 s before 120 µL 6 µg mL^−1^ AO in buffer (37 × 10^−3^ m citric acid, 126 × 10^−3^ m Na_2_HPO_4_·2H_2_O, 150 × 10^−3^ m NaCl, 1.10 × 10^−3^ m EDTA‐2Na·2H_2_O, pH = 6) was added to stain the cells. All chemicals were purchased from Sigma‐Aldrich unless otherwise specified.

*Sperm Acrosome Stain*: The acrosomes of the air‐dried sperm on HA‐modified glass were stained with a solution of 40 µg mL^−1^ lectin PNA from *A. hypogaea* (peanut) conjugate in phosphate‐buffered saline (PBS, pH 7.4, Life Technologies, Canada) and imaged immediately under a spinning disk confocal microscope at a total magnification of 100 × with an excitation wavelength of 647 nm.

*Cell Imaging by Spinning Disk Confocal Microscopy*: After AO staining, sperm were immediately imaged under a spinning disk confocal microscope (Zeiss, AxioObserverZ1 inverted) under a total magnification of 100 ×. Fluorescence images were captured first. The excitation wavelength of the laser source was 488 nm and the bands for the emission filters were set at 500–550 nm and 598–660 nm for green and red emission, respectively. For imaging of green and red fluorescence, the laser intensity and exposure time were always kept at 5% and 200 ms, respectively. The green and red fluorescence intensity of individual sperm was determined by the maximum projected intensity of the sperm. Fluorescent microbeads (6 µm, 488 nm excitation, and 515–660 nm emission, ThermoFisher Scientific) typically used for flow cytometry calibration were used here to verify the consistency of the imaging conditions. Brightfield images were captured afterward with image contrast enhanced (Adobe Lightroom) to better distinguish acrosomal regions.

*Cell Imaging by Scanning Electron Microscopy*: Prior to imaging, the sperm bound to HA glass were fixed with a mixture of methanol and acetic acid (75%:25%, v/v) for 30 min. Once dry, sperm bound to HA glass were coated with Au nanoparticles using an SC7640 High Resolution Sputter Coater (Quorum Technologies) for 15 s at 2.0 kV. SEM imaging was carried out on the Quanta FEI Scanning Electron Microscope.

*Determination of Single‐Sperm DFI*: The DFI values for individual sperm were determined as the ratio of red fluorescence to the total (red + green) fluorescence of the fluorescence images. The red and green fluorescence intensity of each sperm was quantified by a MATLAB program to determine Fragmented DNA and native DNA stainability.

*Machine Learning*: For the linear regression, JMP pro 13 with a five‐fold cross‐validation was used. The stopping rule was Max Validation RSquare with a forward direction. For each test group, all other data were used to train and validate the algorithm. Nonlinear regression analysis was implemented in MATLAB version R2018a (9.4.0) from Mathworks with the Neural Network Toolbox. A two‐layer feed‐forward neural network was used, with a tan‐sigmoid transfer function in the hidden layer with 20 hidden neurons and a linear transfer function in the output layer. The input vectors and target vector were randomly divided into three sets of samples as follows: 65% of data were used for training the algorithm, 10% was used for validating the network performance, and 25% was used to independently test the network. Then this network was applied to an independent test group to check its performance. The neural network was trained with the Levenberg–Marquardt backpropagation function. The training continued until the validation error failed to decrease for 20 iterations. For both linear and nonlinear regressions, the prediction group was either created by using random selected cells across all samples or by using entire individual donor samples, with the rest of cells randomly assigned as training and validation groups. Logistic regression was also performed by JMP pro 13. The output of sperm DNA integrity was classified into two categories according to the rank of Ln(DFI). The input data were randomly divided into three sets with the ratio of 65%, 10%, and 25% for training, validation, and testing, respectively.

*Statistics*: For statistical analyses, an unpaired and two‐tailed Welch's *t*‐test was applied for two‐group comparisons. An analysis of variance (ANOVA) test was applied for groups of more than 3. Significance was determined as *p* < 0.05. Error bars represent the standard error of mean.

## Conflict of Interest

The authors declare no conflict of interest.

## Supporting information

Supplementary

Supplementary

Supplementary
