# Supplementary material for: Prediction of DNA Integrity from Morphological Parameters Using a Single‐Sperm DNA Fragmentation Index Assay
Source: Adv Sci (Weinh). 2019 May 24;6(15):1900712. doi: 10.1002/advs.201900712 (PMC6685501; doi:10.1002/advs.201900712)
Supplement: Supplementary file 1 — Supplementary [file ADVS-6-1900712-s002.pdf]

Copyright WILEY-VCH Verlag GmbH & Co. KGaA, 69469 Weinheim, Germany, 2019.

ADVANCED SCIENCE
Open Access

Supporting Information

for *Adv. Sci.,* DOI: 10.1002/advs.201900712

Prediction of DNA Integrity from Morphological Parameters Using a Single-Sperm DNA Fragmentation Index Assay

*Yihe Wang, Jason Riordon, Tian Kong, Yi Xu, Brian Nguyen, Junjie Zhong, Jae Bem You, Alexander Lagunov, Thomas G. Hannam, Keith Jarvi, and David Sinton**

Copyright WILEY-VCH Verlag GmbH & Co. KGaA, 69469 Weinheim, Germany, 2019.

Supporting Information

Prediction of DNA integrity from morphological parameters using a single-sperm DNA fragmentation index assay

*Yihe Wang, Jason Riordon, Tian Kong, Yi Xu, Brian Nguyen, Junjie Zhong, Jae Bem You, Alexander Lagunov, Thomas G. Hannam, Keith Jarvi and David Sinton**

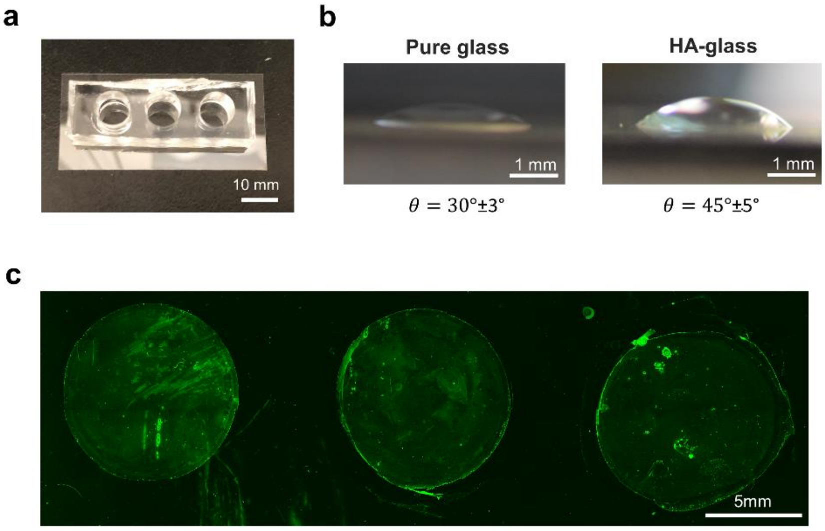

Figure S1. Physical properties of the hylauronic acid (HA) modified glass cover slide. a) The device for preparation of sperm on HA-glass for cell imaging. b) Water contact angle (θ) on a pure glass cover slide and a HA-glass. c) Fluorescence images of a glass cover slide chemically functionalized with fluorescein isothiocyanate (FITC) labeled HA. The HA-slide was washed by plenty of water to ensure removing extra FITC-HA physically absorbed on glass.

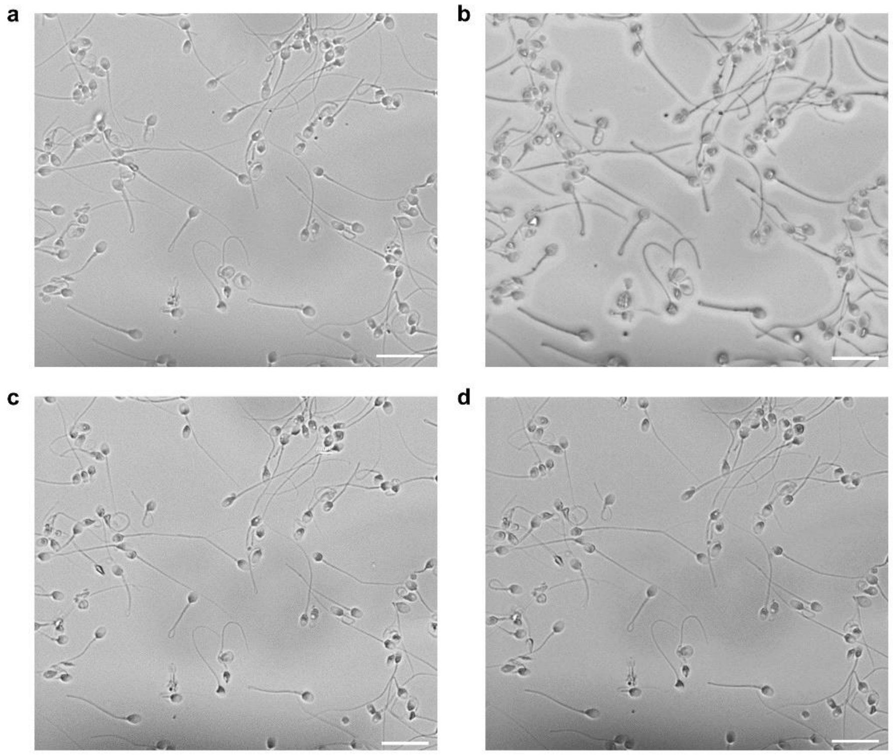

Figure S2. Brightfield images of sperm before and after air-drying and acridine orange (AO) staining. **a)** A brightfield image of sperm before air-drying, **b)** after air-dying, **c)** after adding AO solution, and **d)** one hour after adding the AO solution. Scale bars are 20 µm.

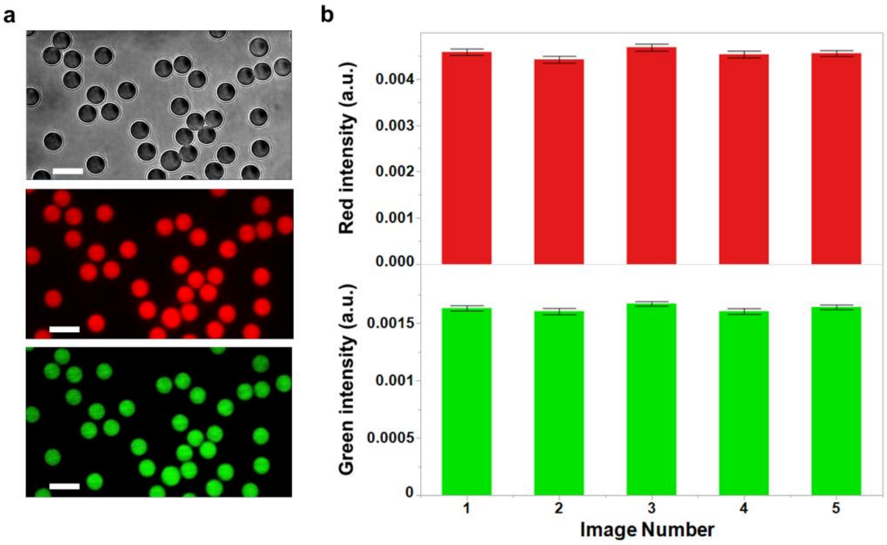

Figure S3. Verification of fluorescence image brightness consistency. a) Fluorescence images of microbeads taken under the same condition as the sperm stained by AO. Scale bars are 10 µm. b) Average fluorescence intensity of microbeads for each image. For all images, $p > 0.05$ using ANOVA

statistical analysis, indicating no significant difference of fluorescence intensity between different images.

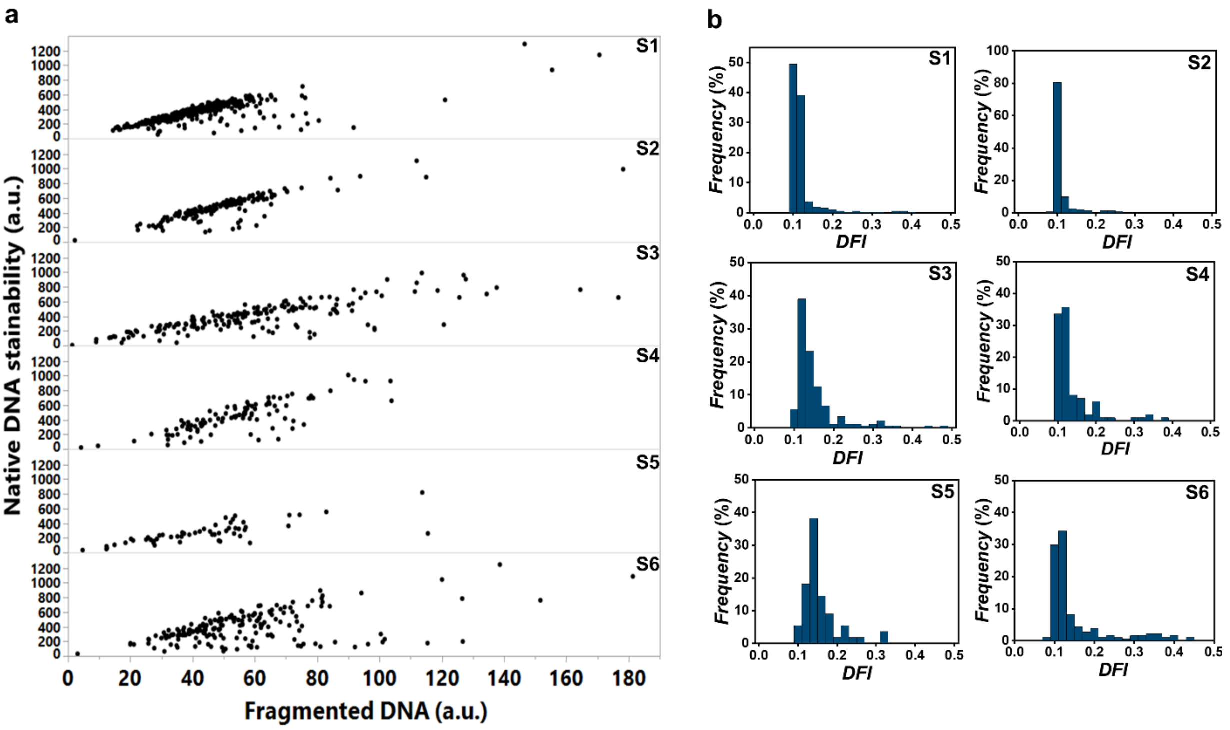

Figure S4. a) Distribution of Native DNA stainability against single sperm fragmented DNA. b) Distribution of single sperm DNA fragmentation Index (*DFI*).

**Predicted *Ln(DFI)* Rank**	**S1**		**S2**		**S3**		**S4**		**S5**		**S6**	
**(%)**	**linear**	**Non-linear**	**linear**	**Non-linear**	**linear**	**Non-linear**	**linear**	**Non-linear**	**linear**	**Non-linear**	**linear**	**Non-linear**
**0-5**	0.078	0.069	0.934	0.696	0.284	0.017	0.999	0.171	0.244	0.105	0.007	0.872
**5-10**	0.043	0.000	0.130	0.343	0.036	0.559	0.993	0.615	0.778	0.768	0.066	0.000
**10-20**	0.000	0.000	0.108	0.014	0.000	0.001	0.376	0.157	0.885	0.668	0.000	0.064
**20-80**	0.012	0.017	0.124	0.035	0.123	0.306	0.045	0.167	0.619	0.863	0.104	0.117
**80-90**	0.340	0.551	0.510	0.350	0.104	0.099	0.021	0.184	0.360	0.965	0.205	0.596
**90-95**	0.013	0.002	0.099	0.450	0.027	0.003	0.281	0.075	0.940	0.968	0.000	0.001
**95-100**	0.002	0.000	0.133	0.011	0.023	0.461	0.193	0.269	0.699	0.497	0.002	0.005
**5-10**	0.043	0.000	0.130	0.343	0.036	0.559	0.993	0.615	0.778	0.768	0.066	0.000

Table S1. *P* values from a *t*-test for the two sets of data: actual *Ln*(*DFI*) in each predicted *Ln(DFI)* rank and all actual *Ln*(*DFI*) by a linear and a non-linear regression for six donor samples (S1-S6).

***Ln(DFI)* Rank**	**Training**		**Validation**		**Test**	
	**Accuracy**	**AUC**	**Accuracy**	**AUC**	**Accuracy**	**AUC**
**0-10% *vs* 10%-100%**	0.891	0.738	0.949	0.716	0.900	0.718
**0-20% *vs* 20%-100%**	0.785	0.748	0.855	0.761	0.815	0.713
**0-30% *vs* 30%-100%**	0.722	0.742	0.761	0.745	0.746	0.741
**0-40% *vs* 40%-100%**	0.669	0.739	0.675	0.711	0.696	0.731
**0-50% *vs* 50%-100%**	0.669	0.755	0.607	0.689	0.712	0.769

**0-60% *vs* 60%-100%**	0.723	0.783	0.744	0.788	0.735	0.784
**0-70% *vs* 70%-100%**	0.792	0.793	0.761	0.788	0.788	0.741
**0-80% *vs* 80%-100%**	0.844	0.795	0.778	0.770	0.854	0.827
**0-90% *vs* 90%-100%**	0.891	0.738	0.949	0.716	0.900	0.718

Table S2. Accuracy and area under the curve (AUC) values for training, validation and test data set for different classifications by sperm *Ln(DFI)* rank.
